# Disparities in Industry Payments to Orthopedic Surgeons: A Retrospective Observational Analysis of Institutional Prestige and Financial Distribution

**DOI:** 10.7759/cureus.82195

**Published:** 2025-04-13

**Authors:** Devon R Pekas, Morgan Pierce, Darren T Hackley, Alexander R Garcia, Parker K Chenault, Mark W Schmitt, Nicholas J Peterman, Chad B Carlson

**Affiliations:** 1 Department of Orthopaedic Surgery, Carilion Clinic, Roanoke, USA; 2 Department of Orthopaedics, University of North Dakota School of Medicine and Health Sciences, Grand Forks, USA; 3 Department of Orthopaedic Surgery, Womack Army Medical Center, Fort Bragg, USA; 4 Department of Orthopaedic Surgery, The Bone & Joint Center, Bismarck, USA

**Keywords:** gini coefficient, healthcare equity, industry payments, orthopedic surgery, royalties

## Abstract

Financial relationships between orthopedic surgeons and industry partners shape innovation and surgical advancements, yet these payments are highly concentrated among a small subset of surgeons. This retrospective observational analysis examines industry payment disparities using the Centers for Medicare & Medicaid Services (CMS) Open Payments Database for 2021, comparing payments between orthopedic surgeons affiliated with U.S. News & World Report (USNWR) top 10 orthopedic centers and those at large academic medical centers in the 25 most populous U.S. states. A total of 1,612 surgeons were included. Industry payments exhibited a right-skewed distribution, with a Gini coefficient of 0.91 for USNWR institutions and 0.93 for state-based academic centers, indicating significant financial stratification. Royalty and licensing fees accounted for 93.25% of payments exceeding $500,000, disproportionately benefiting surgeons at USNWR-ranked institutions. Surgeons at these institutions had 3.01 times higher odds of receiving over $500,000 in payments compared to those at state medical centers (OR = 3.01, 95% CI: 1.50-6.04, p < 0.001). Geographic disparities were also significant, with payments concentrated in states housing top-tier orthopedic programs. These findings highlight the role of institutional prestige in industry compensation and raise ethical considerations regarding equity in financial relationships and access to industry collaborations.

## Introduction

Financial relationships between orthopedic surgeons and industry partners, particularly pharmaceutical and medical device companies, play a critical role in advancing musculoskeletal innovation [1]. These collaborations have led to significant advancements in implants, navigation technologies, and surgical techniques, with royalties and licensing fees serving as primary financial incentives [2]. However, prior research has shown that general payments, industry payments unrelated to research, are highly concentrated among a small subset of surgeons, raising concerns about financial disparities, equitable access to innovation, and potential biases in treatment decisions [3,4].

In response to growing concerns regarding transparency in physician-industry relationships, the Physician Payments Sunshine Act (PPSA) was enacted in 2010 as part of the Affordable Care Act [5,6]. This legislation mandates the public reporting of non-research-related payments exceeding $10 USD to healthcare providers [7]. The Centers for Medicare and Medicaid Services (CMS) Open Payments database, established through this act, provides a comprehensive record of general payments, including consulting fees, royalties, licensing agreements, honoraria, and travel reimbursements [2]. These data allow for critical analyses of financial distributions within medical specialties, including orthopedic surgery [2].

Prior studies have documented that orthopedic surgeons receive some of the highest general payments among all physician specialties, largely driven by consulting fees, royalties, and licensing agreements [3,4]. However, the distribution of these payments is notably unequal. Previous investigations have reported Gini coefficients approaching 0.95, indicating extreme financial stratification, with a small proportion of surgeons receiving the vast majority of payments [3,4]. Furthermore, academic affiliation, geographic location, and institutional prestige have been identified as potential factors influencing access to industry collaborations, though the extent of their impact remains unclear [8,9].

Highly ranked academic medical centers often attract greater financial support from industry due to their established research infrastructure, faculty involvement in technological development, and proximity to major medical industry hubs [10,11]. Conversely, surgeons at lower-resourced institutions may face barriers in accessing similar financial opportunities, limiting their ability to contribute to innovation and participate in industry partnerships [11]. These disparities raise important ethical considerations regarding conflicts of interest, clinical decision-making biases, and the broader implications of financial inequities in surgical innovation [12-14].

This study examines the distribution of general payments among orthopedic surgeons at medical centers of varying prestige. Specifically, it compares payments received by surgeons affiliated with U.S. News & World Report (USNWR) Top 10 orthopedic medical centers to those at large academic medical centers in the most populous U.S. states [15]. By analyzing national payment trends, the study evaluates whether institutional prestige influences financial relationships with industry partners and contributes to disparities in funding distribution. We hypothesized that orthopedic surgeons at USNWR Top 10 institutions would receive significantly higher industry payments than their counterparts at state-based academic centers, with institutional prestige serving as a key predictor of payment concentration.

## Materials and methods

This was an observational, retrospective analysis of orthopedic surgeons listed in the (CMS) Open Payments database for 2021 (January 1, 2021 - December 31, 2021) [16]. To preserve institutional anonymity, medical centers were assigned coded identifiers for reporting, with detailed institutional data provided in the Appendices.

Study population & selection criteria

Two cohorts were examined. Orthopedic surgeons at the 2021 U.S. News & World Report (USNWR) orthopedic medical centers, representing high-prestige institutions [15,17]. Orthopedic surgeons at the largest academic medical centers (determined by hospital bed count) in the 25 most populous U.S. states, representing high-volume, non-elite institutions. This comparison cohort was selected to evaluate how institutional prestige, independent of size and geographic coverage, may influence disparities in industry payments.

If a state’s largest academic medical center overlapped with a USNWR-ranked institution, the most populous state’s center was selected to ensure cohort independence.

Hospital bed counts were obtained from Hospital Management, and state population data was retrieved from the 2020 United States Census [18,19]. Surgeon rosters were sourced from institutional websites and manually cross-referenced in the CMS Open Payments database to extract general payment data [20].

The CMS Open Payments database, established under the Sunshine Act provisions of the ACA, includes public records of general payments, including consulting fees, royalties, licensing agreements, honoraria, and travel reimbursements. This initiative was originally proposed in legislation (S.301) aimed at increasing transparency in industry-physician financial relationships (Appendices) [5,6].

The USNWR rankings were chosen as a benchmark for institutional prestige, as such rankings may influence industry relationships and access to funding [17].

Eligibility criteria

Included participants were orthopedic surgeons with a valid National Provider Identifier (NPI) affiliated with a qualifying institution’s main campus in 2021. Exclusion criteria encompassed non-physician healthcare providers, physicians without Accreditation Council for Graduate Medical Education (ACGME)-accredited orthopedic surgery residency training, orthopedic surgery trainees, and surgeons primarily affiliated with satellite or non-main campus locations. Duplicate NPI entries and payment records not classified as general payments were also excluded. Surgeon affiliations were manually verified against institutional rosters to ensure cohort accuracy.

A total of 1,612 orthopedic surgeons met the inclusion criteria: 821 from USNWR institutions and 791 from state-based academic centers.

Statistical analysis

Data analysis was performed using Python 3.12, with statistical procedures conducted via NumPy, StatsModels, and Pandas libraries. The Shapiro-Wilk test assessed normality of continuous variables. For group comparisons, Student’s t-test was used for parametric data and the Mann-Whitney U test for non-parametric data. One-way ANOVA and Kruskal-Wallis tests were used for multi-group comparisons, depending on distribution. Categorical variables were evaluated using Chi-square tests, with Fisher’s exact test applied when expected counts were small.

To evaluate industry payment disparities, a Lorenz curve was generated to illustrate the distribution of payments across institutions, and Gini coefficients were calculated separately for USNWR Top 10 and state academic centers to quantify financial inequality. A Mann-Whitney U test was used to compare overall payment distributions between the two cohorts. Odds ratios (ORs) were calculated to assess the likelihood of surgeons receiving payments exceeding $500,000 based on institutional affiliation. A multiple linear regression model was constructed to evaluate the association between institutional prestige and total industry payments, adjusting for faculty size and state population. Geographic disparities were examined using a Kruskal-Wallis test, followed by post-hoc pairwise comparisons across states to identify regional differences.

All statistical tests were two-tailed, with p < 0.05 considered statistically significant.

While this analysis did not adjust for potential confounders such as surgeon experience, subspecialty, academic productivity, or leadership roles, due to data limitations, these factors are likely to contribute to payment variation. Additionally, as this was a retrospective observational study, causal inferences cannot be made.

## Results

General payment distribution to orthopedic surgeons

**Figure 1:**
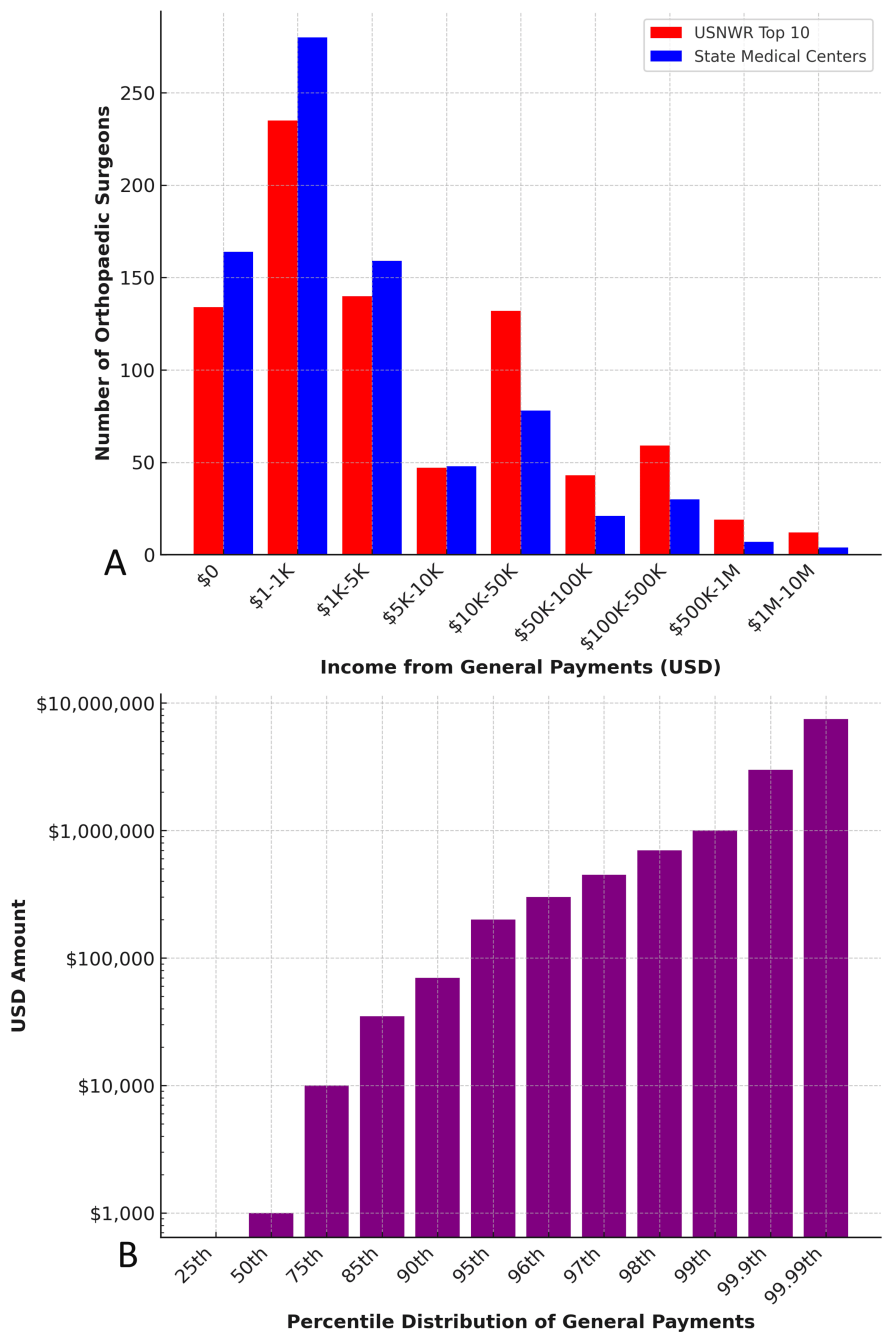
Distribution of General Payments to Orthopedic Surgeons by Institutional Affiliation and Percentile Ranking (2021) A: Histogram comparing the number of orthopedic surgeons at USNWR Top 10 and State medical centers across income brackets from general payments. B: Percentile distribution of total general payments received by orthopedic surgeons, showing an exponential increase in payments among top earners. USNWR: U.S. News & World Report

Among USNWR Top 10 hospitals, 31 surgeons received payments exceeding $500,000, compared to 11 at state hospitals (Table 1, Figure 1A). The proportion of surgeons receiving over $50,000 was significantly higher in Top 10 institutions, underscoring the concentration of industry relationships at elite centers. Surgeons at USNWR Top 10 institutions had 3.01 times higher odds of receiving payments exceeding $500,000 compared to those at state medical centers (OR = 3.01, 95% CI: 1.50-6.04, p < 0.004).

**Table 1:** Distribution of General Payments to Orthopedic Surgeons by Institutional Category (2021, USD) USNWR: U.S. News & World Report

Payment Bracket (USD)	Number of Orthopedic Surgeons at Largest State Medical Centers	Number of Orthopedic Surgeons at USNWR Top 10 Medical Centers
$0	164	134
$1-$1K	280	235
$1K-$5K	159	140
$5K-$10K	48	47
$10K-$50K	78	132
$50K-$100K	21	43
$100K-$500K	30	59
$500K-$1M	7	19
$1M-$10M	4	12

Industry payments exhibited a strong right-skew, favoring higher earners (Table 2, Figure 1B). The median payment was $975.37, while the 90th percentile received $67,776.72. Payments escalated sharply beyond the 95th percentile, with the 99th percentile receiving $951,861.74 and the 99.99th percentile reaching $7.45 million. Most surgeons fell within the $0-$5K payment range, though USNWR Top 10 surgeons had a significantly greater proportion in the >$10K bracket (p < 0.001).

**Table 2:** Percentile Distribution of General Payments to Orthopedic Surgeons (2021, USD)

Percentile Rank of General Payments	Payment Received (USD)
5^th^	$0
10^th^	$0
15^th^	$0
20^th^	$15.95
25^th^	$53.23
30^th^	$108.28
35^th^	$178.48
40^th^	$303.76
45^th^	$503.60
50^th^	$975.37
55^th^	$1,316.82
60^th^	$2,175.68
65^th^	$3,566.97
70^th^	$5,782.66
75^th^	$10,038.34
80^th^	$17,643.49
85^th^	$34,402.32
90^th^	$67,776.72
95^th^	$196,777.45
96^th^	$292,314.78
97^th^	$422,663.92
98^th^	$655,525.14
99^th^	$951,861.74
99.9^th^	$2,932,102.42
99.99^th^	$7,449,162.29

Nature of industry payments

**Figure 2:**
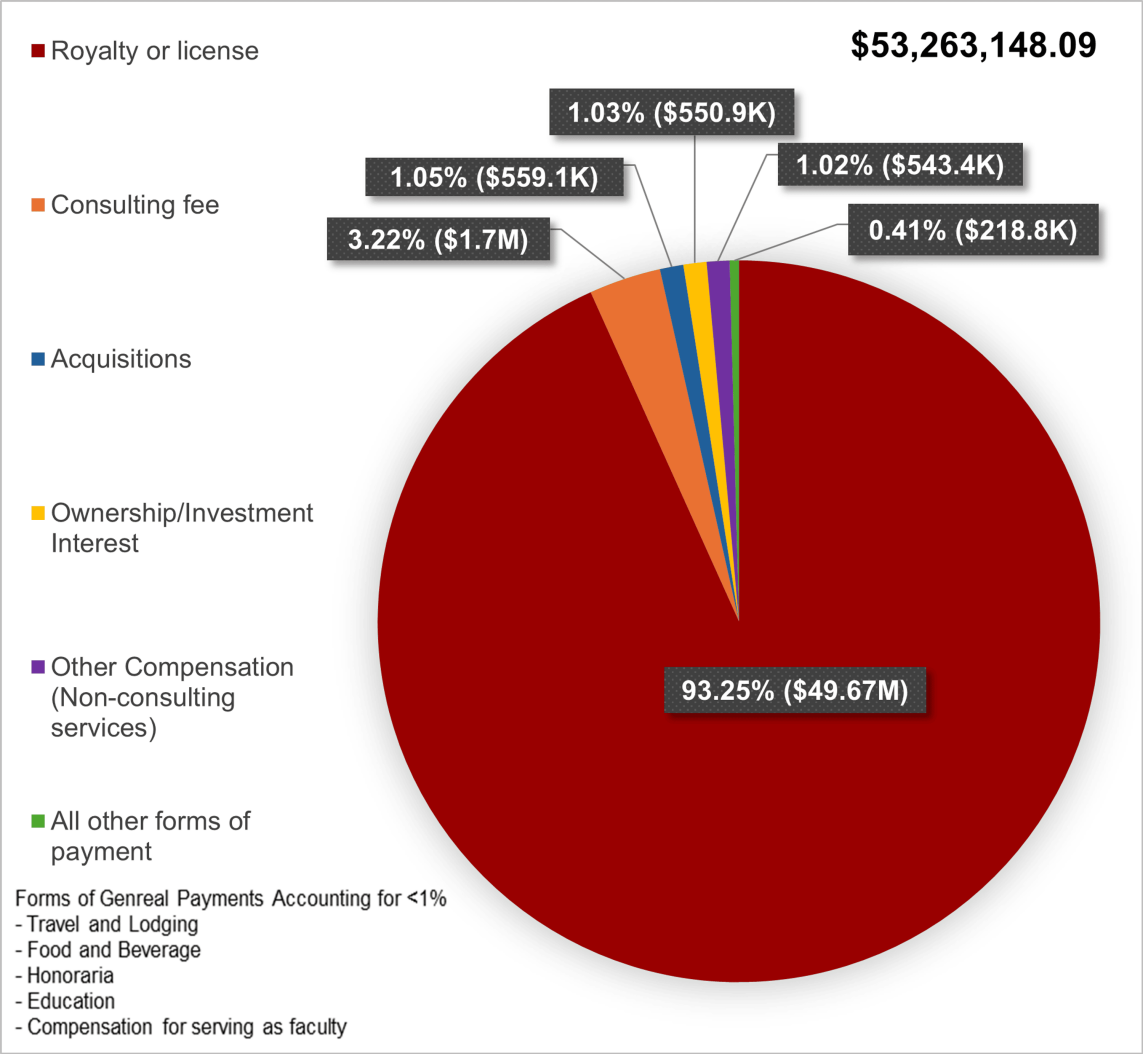
Proportion of General Industry Payments to Orthopedic Surgeons by Payment Type (2021, USD) Pie chart displaying the distribution of industry payments by payment type. The largest proportion of payments consists of royalty or license fees, followed by consulting fees. Other payment categories include acquisitions, ownership or investment interests, compensation for services other than consulting and all other forms of payment.

Industry payments were primarily composed of royalty or license fees (93.25%; Figure 2). Consulting fees accounted for 3.22%, while acquisitions, ownership interests, and other compensation types comprised 3.51%, emphasizing the dominance of intellectual property agreements.

Industry payment concentration and inequality

**Figure 3:**
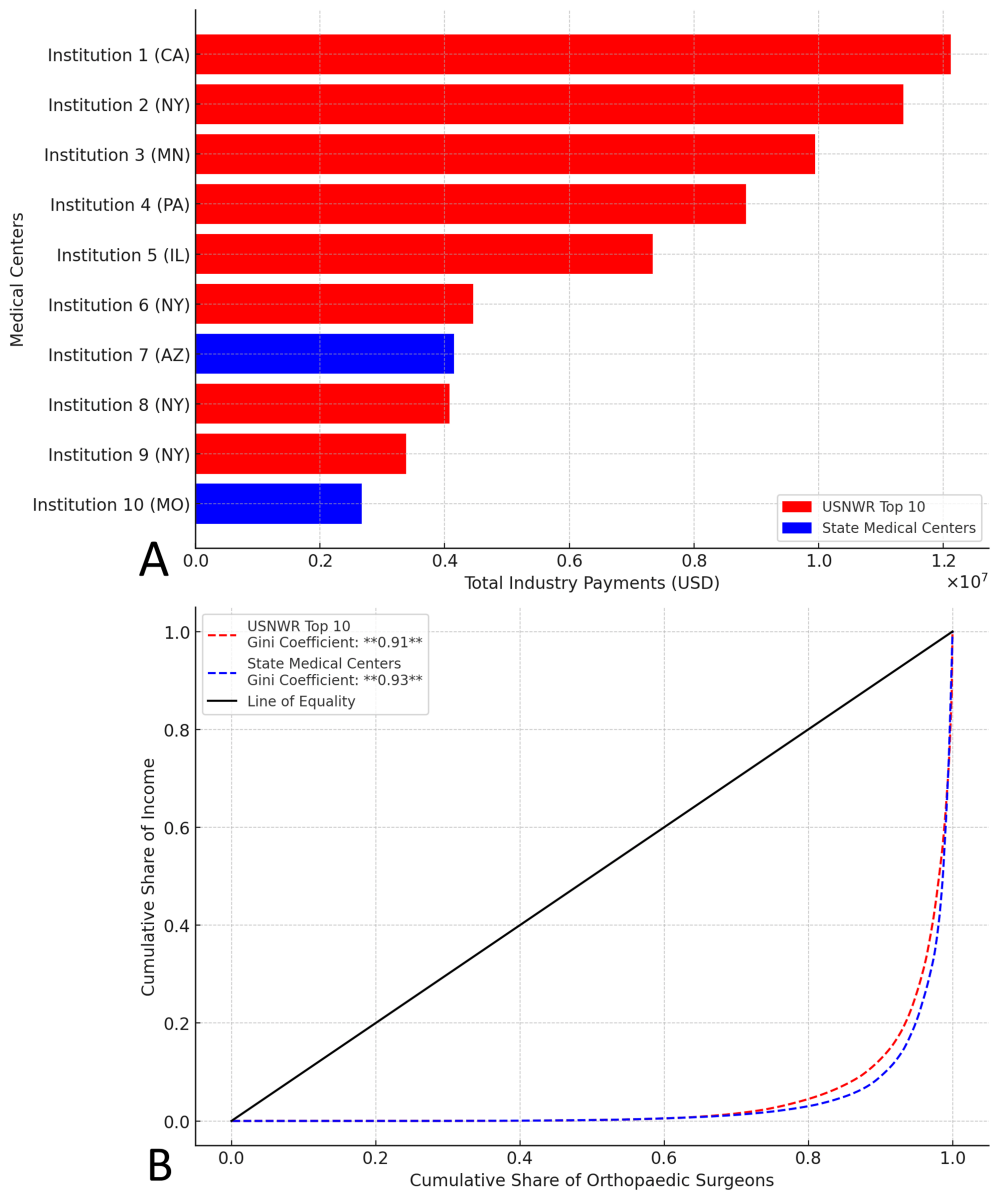
Distribution and Inequality of Industry Payments to Orthopedic Surgeons by Institution (2021) A: Total industry payments to orthopedic surgeons at USNWR Top 10 and state medical centers. Institutions are coded for anonymity, with corresponding states indicated in parentheses. B: Lorenz curve analysis of industry payment distribution among orthopedic surgeons. The Gini coefficient indicates substantial payment inequality, with USNWR Top 10 and state medical centers. The line of equality represents perfect income distribution. USNWR: U.S. News & World Report

USNWR Top 10 institutions received significantly higher total industry payments than state medical centers (Figure 3A). A ranking of institutions by total payments showed a steep decline beyond top-ranked hospitals, indicating a high concentration within a few prestigious centers.

A Lorenz curve analysis illustrated substantial inequality in payment distribution (Figure 3B). The Gini coefficient for USNWR Top 10 institutions was 0.91, while state medical centers exhibited slightly greater inequality at 0.93.

Faculty size and total industry payments

**Figure 4:**
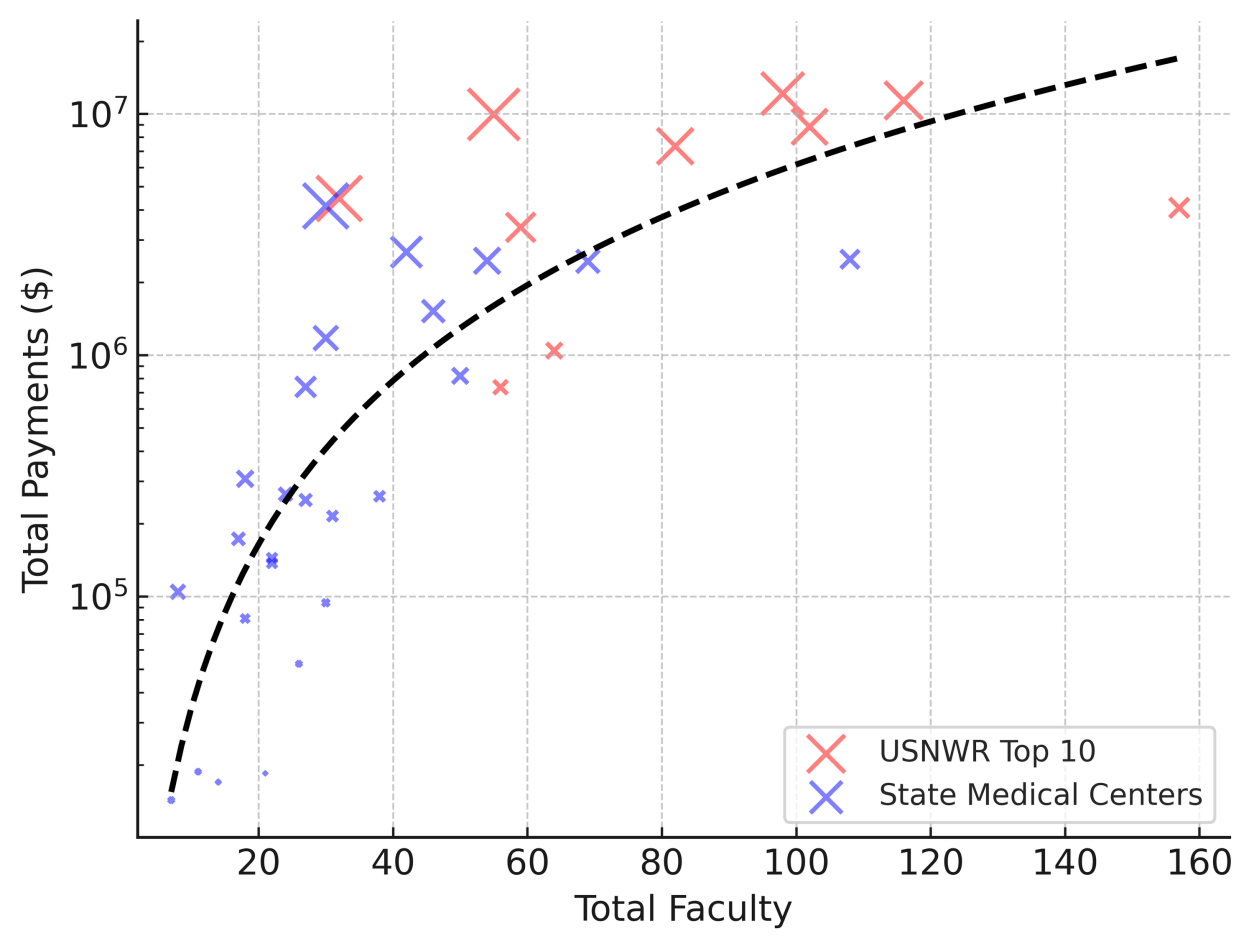
Relationship between Faculty Size and Total Industry Payments in Orthopedic Surgery (2021) Scatter plot showing total industry payments ($USD) versus faculty size at USNWR Top 10 and state medical centers. “X” size corresponds to total payments per institution. The black dashed line represents the fitted regression curve, illustrating a non-linear (logarithmic) relationship between faculty size and industry payments. USNWR: U.S. News & World Report

A regression analysis confirmed that faculty size significantly predicted total payments (β = 38.54K, SE = 15.4K, t = 2.510, p = 0.017, 95% CI: [7.23K-69.9K]; Table 3, Figure 4). USNWR prestige was also a significant predictor (β = 3.815M, SE = 1.12M, t = 3.409, p = 0.002, 95% CI: [1.53M-6.1M]), indicating its role in payment concentration. The proportion of payments received by the highest-paid surgeon at an institution was not a significant predictor (β = 17.09K, SE = 23K, t = 0.744, p = 0.463).

**Table 3:** Regression Results: Predictors of Institutional Financial Outcomes USNWR: U.S. News & World Report

Predictor Variable	Coefficient (β)	Standard Error	t-Value	p-Value	95% Confidence Interval
Intercept (Constant)	-1.23M	1.43M	-0.860	0.396	(-4.15M, 1.69M)
USNWR Prestige (1 = Top 10)	3.815M	1.12M	3.409	0.002	(1.53M, 6.1M)
Total Faculty	38.54K	15.4K	2.510	0.017	(7.23K, 69.9K)
Highest Earner Share (%)	17.09K	23K	0.744	0.463	(-29.8K, 63.9K)

Geographic payment distribution

**Figure 5:**
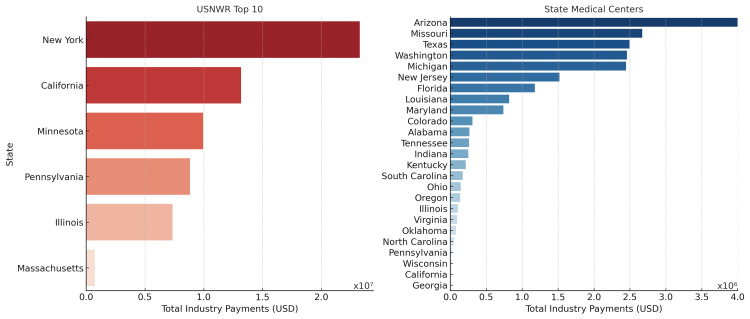
State-Level Distribution of Industry Payments to Orthopedic Surgeons by Institutional Category (2021) State-level distribution of total industry payments ($USD) to orthopedic surgeons at USNWR Top 10 and state medical centers. Bars represent the aggregated total payments per state, highlighting geographic disparities in industry compensation. Payments were highest in New York, California, and Minnesota for USNWR Top 10 medical centers and in Arizona, Missouri, and Texas for state medical centers. USNWR: U.S. News & World Report

Industry payments varied significantly by state (Kruskal-Wallis, p < 0.001; Figure 5). Among USNWR Top 10 institutions, total industry payments were highest in New York ($21.89 million), California ($13.16 million), Minnesota ($9.94 million), Pennsylvania ($8.83 million), Illinois ($7.33 million), and Massachusetts ($0.74 million). Among state medical centers, total payments were highest in Arizona ($4.15 million), Missouri ($2.67 million), Texas ($2.50 million), Washington ($2.46 million), and Michigan ($1.52 million). Pairwise post hoc comparisons revealed that USNWR Top 10 institutions had significantly higher payments than state medical centers across all states (p < 0.001). 

## Discussion

This study analyzes industry payments to orthopedic surgeons, highlighting significant disparities based on institutional prestige and geographic location. Prior research shows that industry payments to orthopedic surgeons are highly concentrated, with reported Gini Coefficients as high as 0.95 [4]. Our findings align with this trend, with Gini coefficients of 0.91 for USNWR medical centers and 0.93 for state medical centers, indicating persistent financial stratification. Notably, 2% of surgeons receive the majority of funds, while 50.4% of surgeons received payments below $1,000, underscoring the unequal distribution of industry payments.

These findings indicate persistent financial stratification, reinforcing concerns about structural inequities in industry compensation. Such disparities may influence research opportunities, professional advancement, and the dissemination of new surgical technologies, disproportionately benefiting high-profile institutions while limiting access to innovation at lower-resourced centers [8,10].

A key finding of this study is that royalties and licensing fees dominate industry payments, highlighting the critical role of intellectual property in surgeon-industry relationships. Prior research estimates that these payments account for nearly 70% of total industry compensation to orthopedic surgeons, reflecting the field’s reliance on surgeon-driven innovation [1,21]. Similarly, royalties and licensing fees made up 69% of industry payments to orthopedic surgeons, and in a broader five-year review, they accounted for 68.9% of general payments [3,4]. Our findings reinforce this, with royalty and licensing fees comprising 93.25% of payments. However, these payments are disproportionately concentrated at elite institutions and among a small subset of high-profile surgeons, potentially limiting the widespread adoption of new surgical technology [11]. These ethical concerns include conflicts of interest, biases in clinical decision-making, and potential erosion of patient trust [2]. Given that orthopedic surgeons are among the highest-compensated specialists, ongoing scrutiny is essential to ensure financial incentives do not compromise patient care or equitable access to innovation.

The geographic variation observed in this study aligns with prior findings that industry payments are unevenly distributed across regions. Open payments data analyses have shown that funding is often concentrated in states with prestigious medical institutions. Studies by Choudhry et al. and Murayama et al. demonstrate that payments are disproportionately allocated to regions with top-ranked academic centers with states such as New York and California consistently receiving the highest total payments [22,23]. Similarly, Garstka et al. identified significant geographic disparities in industry payments, showing that certain states receive substantially higher mean payments, further highlighting the influence of location on funding distribution [24]. Our findings confirm these trends, demonstrating that industry payments remain highest in states with top-ranked orthopedic medical centers, emphasizing the influence of institutional prestige and geography on financial relationships with industry partners.

The translational impact of these disparities is considerable. When royalty payments are concentrated among a select group of surgeons at elite institutions, the broader adoption of novel surgical techniques may be hindered, potentially contributing to uneven access to surgical innovation [11]. This dynamic may disadvantage surgeons at lower-resourced hospitals in terms of training opportunities, access to emerging technologies, and participation in industry-driven research [3,10]. The persistence of such disparities may further widen the gap in professional advancement [11,25]. To address these inequities, institutions and policymakers may consider exploring interventions, such as structured mentorship programs and regional innovation networks, which have been successfully modeled in oncology and cardiology [26-28]. Combining these initiatives with equity-based funding mechanisms and investments in translational research infrastructure at non-USNWR centers could improve access to industry collaborations and promote equitable distribution of financial resources [26,29].

While transparent reporting of financial relationships is essential for maintaining public trust, patient perception remains mixed [21,30,31]. Hwong et al. identified potential negative views toward physicians receiving substantial industry payments, whereas systematic reviews by Fadlallah et al. demonstrated that patients and the general public were generally supportive or neutral regarding surgeon-industry collaborations [30,31]. Similarly, Camp et al. reported that transparency initiatives, such as the Sunshine Act, have not consistently improved patient trust, suggesting that disclosure alone has minimal or no impact [2,6,21]. The concerns that led to the Sunshine Act were first introduced in legislative proposals such as S.301, highlighting the need for publicly accessible financial disclosures [5,24]. These findings emphasize that transparency initiatives must be complemented by equity-driven policies to address structural inequalities, promote fair resource distribution, and enhance patient-centered care [12-14].

This study has several limitations. The CMS Open Payments database relies on self-reported data from manufacturers, introducing the potential for underreporting, misclassification, or incomplete disclosures [7]. Prior studies have identified selection bias and discrepancies in reported data across specialties and geographic regions, further limiting the accuracy of findings [32]. Additionally, certain financial interactions, such as indirect payments through third parties, stock options, or separately categorized research funding, may not be fully captured, potentially underestimating the scope of industry collaborations. The study’s focus on academic medical centers also limits the generalizability to private-practice orthopedic surgeons, who represent a substantial proportion of the field and may have differing industry affiliations and payment structures. Finally, this analysis also does not control for potential confounding factors such as surgeon experience, subspecialty focus, research productivity, or leadership roles, all of which may influence industry payment distribution. Future research should incorporate a broader range of practice settings, surgeon-reported perspectives on barriers to industry collaboration, and account for potential selection bias and reporting inconsistencies across specialties and geographic regions.

## Conclusions

This study demonstrates substantial disparities in industry payments to orthopedic surgeons, with institutional prestige and geography emerging as significant contributors to financial concentration. Royalties and licensing fees accounted for the majority of high-value payments and were disproportionately directed toward surgeons at USNWR-ranked institutions. While these findings underscore structural inequities, the analysis did not adjust for potential confounders such as surgeon experience, subspecialty, academic productivity, or leadership roles, factors likely influencing payment distribution. These limitations are acknowledged and warrant further study. Transparency alone is insufficient; efforts to promote equitable access to industry collaboration, particularly at non-elite centers, are essential to ensuring broader participation in innovation and maintaining public trust in the orthopedic profession.

## Appendices

**Table 4:** Industry Payments to Academic Surgeons: Summary Statistics from the U.S. News World Report Top 10 Orthopaedic Medical Centers (2021)

Program	Total Faculty Count	Total Industry Payments (USD)	Mean Payment per Surgeon (USD)	Std. Dev. of Payments (USD)	Payment Range (USD)	Faculty with No Industry Payment (%)	Faculty with < $500 (%)	Faculty with < $1,000 (%)	Highest Earner Share of Institutional Total (%)
U.S. News World Report Top 10 Medical Centers									
Cedars-Sinai Medical Center	98	$12,120,634	$123,680	$845,624	$0 - $8,304,568	8.16%	44.90%	51.02%	68.52%
Hospital for Special Surgery	116	$11,356,994	$97,905	$329,880	$0 - $2,894,135	22.41%	32.76%	34.48%	25.48%
Mayo Clinic	55	$9,941,620	$180,757	$491,312	$0 - $2,517,386	23.64%	36.36%	41.82%	25.32%
Rothman Orthopaedics (Thomas Jefferson U.)	102	$8,833,780	$86,606	$235,658	$0 - $1,361,126	10.78%	43.14%	50.98%	15.41%
Midwest Orthopaedics (Rush U.)	82	$7,335,719	$114,621	$285,064	$0 - $1,578,184	12.50%	25.00%	29.69%	21.51%
New York-Presbyterian (Columbia & Cornell)	32	$4,457,280	$139,290	$534,524	$0 - $2,991,487	9.38%	25.00%	52.00%	67.11%
NYU Langone Orthopaedic Hospital	157	$4,076,795	$25,967	$92,698	$0 - $945,142	20.38%	46.50%	49.68%	23.18%
Mount Sinai Hospital	59	$3,384,317	$57,361	$217,668	$0 - $1,343,106	11.86%	47.46%	52.54%	39.69%
Massachusetts General Hospital	56	$735,915	$13,141	$39,923	$0 - $244,347	23.21%	41.07%	51.79%	33.20%
Santa Monica-UCLA Orthopaedic Hospital	64	$1,043,417	$12,725	$29,913	$0 - $200,137	14.63%	40.24%	43.90%	19.18%

**Table 5:** Industry Payments to Academic Surgeons: Summary Statistics from the Largest Academic Medical Centers in the 25 Most Populous U.S. States (2021)

Program	Total Faculty Count	Total Industry Payments (USD)	Mean Payment Per Surgeon (USD)	Std. Dev. of Payments (USD)	Payment Range (USD)	Faculty With No Industry Payment (%)	Faculty With < $500	Faculty With < $1,000	Highest Earner Share of Institutional Total (%)
Largest Academic Medical centers in the 25 Most Populous States									
Banner University Medical Center	30	$4,149,806.50	$138,326.88	$357,393.79	$0- $1,442,402.93	13.33%	33.33%	43.33%	34.76%
Barnes Jewish Hospital	42	$2,672,841.97	$63,639.09	$214,252.91	$0- $1,356,075.62	14.29%	35.71%	40.48%	50.74%
Houston Methodist	108	$2,495,194.39	$23,103.65	$102,452.23	$0-$856,323.88	13.89%	47.22%	50.93%	34.32%
Swedish Medical Centre-First Hill Campus	54	$2,459,581.43	$45,547.80	$158,152.90	$0- $809,631.63	35.19%	61.11%	62.96%	32.92%
Beaumont Hospital-Royal Oak	69	$2,446,892.61	$35,462.21	$112,256.71	$0-$693,814.62	14.49%	50.72%	57.97%	28.35%
Hackensack University Medical Center	46	$1,520,895.08	$33,062.94	$181,194.08	$0- $1,222,308.08	15.22%	50.00%	58.70%	80.37%
Orlando Health Orlando Regional Medical Center	30	$1,176,137.76	$39,204.59	$159,939.69	$0-$875,246.69	3.00%	23.33%	33.33%	74.42%
Our Lady Of The Lake Regional Medical Centre	50	$820,016.02	$16,400.32	$54,324.35	$0- $306,789.07	8.00%	56.00%	70.00%	37.41%
The John Hopkins Hospital	27	$738,100.77	$27,337.07	$89,158.35	$0- $447,136.73	25.93%	55.56%	59.26%	60.58%
Presbyterian St. Luke's Medical Center	18	$306,977.41	$17,054.30	$35,719.59	$0- $128,973.47	11.11%	38.89%	50.00%	42.01%
University Of Alabama-Birmingham Hospital	24	$265,372.06	$11,057.17	$34,016.51	$0- $166,741.31	8.00%	45.83%	50.00%	62.83%
Vanderbilt Health	38	$259,893.53	$6,839.30	$18,486.67	$0- $83,424.99	23.68%	52.63%	60.53%	32.10%
Indiana University Health	27	$250,869.72	$9,291.47	$24,013.04	$0- $112,976.34	18.52%	55.56%	66.67%	45.03%
Norton Hospital	31	$215,208.12	$6,942.20	$23,399.52	$0- $127,246.25	32.26%	58.07%	64.52%	59.13%
Medical University Of South Carolina Health University Hospital	17	$173,126.41	$10,183.91	$23,133.07	$0- $91,150.53	35.29%	52.94%	64.71%	52.65%
Ohio State University Wexner Medical Center	22	$144,126.18	$6,551.19	$9,895.22	$0- $35,235.41	36.36%	45.45%	45.45%	24.45%
Oregon Health And Science University	22	$137,405.27	$6,245.69	$15,378.28	$0- $72,544.70	32.36%	45.45%	45.45%	52.80%
University Of Illinois Medical Center	8	$104,489.57	$13,061.20	$28,045.83	$0- $81,555.52	25.00%	37.50%	37.50%	78.05%
Virginia Commonwealth University Medical Center	30	$94,071.34	$3,135.71	$4,524.38	$0- $16,631.78	23.33%	46.67%	50.00%	17.68%
Oklahoma University Medical Center	18	$80,950.19	$4,497.23	$12,605.72	$0- $52,326.41	16.67%	50.00%	66.67%	64.64%
University Of North Carolina Medical Center	26	$52,563.26	$2,021.66	$7,562.69	$0- $38,808.40	42.31%	69.23%	69.23%	73.83%
Temple University	11	$18,776.68	$1,706.97	$3,328.70	$0- $10,021.60	54.55%	72.73%	72.73%	53.37%
Aurora St. Luke’s Medical Center	21	$18,479.06	$879.96	$2,203.62	$0- $9,976.39	38.10%	71.43%	71.43%	53.99%
Santa Clara Valley Medical Center	14	$16,998.32	$1,214.17	$1,841.03	$0- $6,375.82	21.43%	57.14%	57.14%	37.51%
Grady Memorial Hospital	7	$14,314.68	$2,044.95	$2,582.88	$75.56- $5,863.12	0.00%	57.14%	57.14%	40.96%

**Table 6:** General Payment Definitions According to the Centers for Medicare and Medicaid (CMS)

Payment Type	Definition
Acquisitions	Buyout payments made to covered recipients who have ownership interest in a company that has been acquired.
Charitable Contributions	A payment or transfer of value made to a tax-exempt organization. Excludes payments better described by another category.
Compensation for Serving as Faculty or Speaker for a Non-Accredited and Non-Certified Continuing Education Program	Compensation for non-accredited/non-certified program speaking. Applicable for Program Years 2013–2020.
Compensation for Serving as Faculty or Speaker for Medical Education Program	Beginning 2021, includes both accredited/certified and non-accredited/non-certified continuing education programs.
Consulting Fee	Payment for providing expertise or advice on medical products or treatments, usually under a formal agreement.
Current or Prospective Ownership or Investment Interest	Includes both current and potential ownership/investment interests held by physicians or teaching hospitals.
Debt Forgiveness	Forgiving the debt of a covered recipient, physician owner, or their immediate family.
Education	Payments for educational activities such as classes, textbooks, or medical journals.
Entertainment	Includes recreational, cultural, or sporting event attendance.
Food and Beverage	Payments for meals and drinks.
Gift	Items of value provided that do not fit another specific payment category.
Grant	A payment supporting a specific cause or activity by the recipient.
Honoraria	Compensation for brief, one-time services, usually without a set fee.
Long-Term Medical Supply or Device Loan	Loan of medical supplies or devices for 91 days or more.
Medical Education Faculty/Speaker Compensation	Compensation for teaching or speaking roles in medical education.
Royalty or License	Payments based on product sales using the physician’s intellectual property.
Space Rental or Facility Fees	Facility rental fees; applies only to teaching hospitals.
Travel and Lodging	Reimbursement for travel-related expenses, including hotel, airfare, mileage, and taxis.
